# Adaptive Object Tracking via Multi-Angle Analysis Collaboration

**DOI:** 10.3390/s18113606

**Published:** 2018-10-24

**Authors:** Wanli Xue, Zhiyong Feng, Chao Xu, Zhaopeng Meng, Chengwei Zhang

**Affiliations:** 1 School of Computer Science and Technology, Tianjin University, Tianjin 300350, China; xuewanli@tju.edu.cn; 2 School of Computer Software, Tianjin University, Tianjin 300350, China; zyfeng@tju.edu.cn (Z.F.); mengzp@tju.edu.cn (Z.M.); 3 Shenzhen Research Institute of Tianjin University, Shenzhen 518000, China; 4 Information Science and Technology College, Dalian Maritime University, Dalian 116026, China; chenvy@tju.edu.cn

**Keywords:** visual tracking, multi-angle analysis, multi-dimensional state–action space, reinforcement learning, collaboration

## Abstract

Although tracking research has achieved excellent performance in mathematical angles, it is still meaningful to analyze tracking problems from multiple perspectives. This motivation not only promotes the independence of tracking research but also increases the flexibility of practical applications. This paper presents a significant tracking framework based on the multi-dimensional state–action space reinforcement learning, termed as multi-angle analysis collaboration tracking (MACT). MACT is comprised of a basic tracking framework and a strategic framework which assists the former. Especially, the strategic framework is extensible and currently includes feature selection strategy (FSS) and movement trend strategy (MTS). These strategies are abstracted from the multi-angle analysis of tracking problems (observer’s attention and object’s motion). The content of the analysis corresponds to the specific actions in the multidimensional action space. Concretely, the tracker, regarded as an agent, is trained with *Q*-learning algorithm and ϵ-greedy exploration strategy, where we adopt a customized rewarding function to encourage robust object tracking. Numerous contrast experimental evaluations on the OTB50 benchmark demonstrate the effectiveness of the strategies and improvement in speed and accuracy of MACT tracker.

## 1. Introduction

Vision sensors, especially ordinary cameras, are a direct source of computer vision information. Visual tracking which gets the object position quickly and accurately in a continuous video sequence is an important topic in visual sensors research. During the tracking process, many challenges from the object itself and its surroundings need to be addressed, such as illumination variation, scale variation, occlusion, fast motion, background clutters, low resolution, deformation, in-plane rotation and so on [1]. Furthermore, the tracking system is broken into multiple constituent components: motion model, feature extractor, observation model, model updater and ensemble post-processor [2].

Many current research methods have performed well in tracking research, but most of them benefit from powerful deep neural networks and excellent machine learning methods; the former lacks reliable explanation, and the latter is only a method application. Tracking research should return to its essence that computer vision tracking is a simulation of human visual tracking.

To solve the above challenges, first, we need to analyze the tracking process from the perspective of human vision. For instance, we need to concentrate on a pedestrian (wearing a white shirt and black pants), and he walks alone from the forest to the crowd on the side of the road. Tracking in the forest is easy because there are no interfering objects similar to the pedestrian. At this time, we do not have to pay attention to the surrounding background, but only distinguish the target from the color. In addition, most of the surrounding objects are stationary. However, when the pedestrian enters the crowd, tracking becomes difficult, because interference (other people wearing the same color of clothing) appears. At this time, to distinguish between the target and surrounding interference, we need to pay attention to more details, such as tall or short, fat or thin, and individual trajectories.

By carefully analyzing the above example, we find that humans analyze the tracking process from different angles. These angles are formed by specific changes in the object and background during the tracking process. In the above case, on the one hand, during the entire tracking process, we will constantly adjust the focus according to the environment changes from the perspective of subjective observers, for example, from the forest to the crowd, our observation focus shifts from color to body shape. On the other hand, from the perspective of the objective pedestrian himself, the object shows different states due to its own changes (such as object deformation, movement trajectory changes, etc.).

In the tracking process, different observers, targets, and application scenarios will form multiple different perspectives. This multi-angle analysis is the key point that tracking research is different from other research, such as object detection, scene analysis, etc. More critically, we believe that the thinking process should be abstracted and modeled to assist in tracking research. To facilitate verification, in this paper, we only analyze the tracking problem from the cooperation of two angles: the observer’s attention to the object and the object’s motion trajectory.

First, the observer’s attention angle shows the change in the tracker’s focus on the object during the tracking process. The most obvious is the object’s features such as color, texture, structure and so on. The intuition is that, as the environment and object change during the tracking process, the salient features of the object are constantly being replaced. In this paper, we have selected three representative features: raw grayscale, raw color and Histogram of Oriented Gradient (HOG) [3]. Obviously, in the simplest tracking environment, the grayscale feature can complete the tracking task; when the target and the surrounding background color are different, the color feature can achieve the best performance; and, in the more complex environment, HOG feature is the right choice. In fact, the replacement of these features reflects the observer’s process of attention change. We prefer to use raw color and raw grayscale features because they have lower computational complexity than HOG. However, in some cases, for example, in the *football* sequence, the frames are grayscale and there are many similar interferers around the object.), raw color and raw grayscale features tend to cause tracking drift. Under these circumstances, although the HOG has high computational complexity, we still use HOG as the last choice since it has the best overall performance among these three features [2]. Specifically, we use reinforcement learning to get the feature selection strategy for guiding the above feature adjustment.

Second, different from object detection research, the object is moving and changing during the tracking process. Thus, it is critical to analyze tracking problem from the object’s motion trajectory perspective. However, since most video sequences have the following characteristics in tracking research: the reference object is constantly changing and the camera parameters are unknown. It is very difficult to directly find the trajectory of the object, so we propose a description method based on the relative change trend of the object in the current environment called the movement trend strategy which is realized by reinforcement learning. This strategy can predict where the next frame object may appear, thus assisting tracking in the motion model.

To address these challenges, we present a strategic framework to guide and improve the ordinary tracking framework in Section 3.2. In detail, we define the tracking process as a Markov Decision Process (MDP). We use frame as state object, and each frame contains two states: tracking success and tracking failure. It is worth noting that, based on an analysis of the observer’s attention change and object motion, we correspond these thoughts to the multidimensional action space: observer’s attention changes to feature selection; and object’s motion to movement trend. Therefore, the action space of the MDP contains two sets of independent actions: features (HOG, raw color and raw grayscale) and directions (up, down, left, and right). Specifically, in our strategic framework, we use *Q*-learning and ϵ-greedy exploration algorithms to obtain the optimal return of different actions in each state, thus forming corresponding strategies. Furthermore, we use strategies that include feature selection and movement trends, to assist and guide the basic tracking framework which is introduced in Section 3.1.

**Figure 1:**
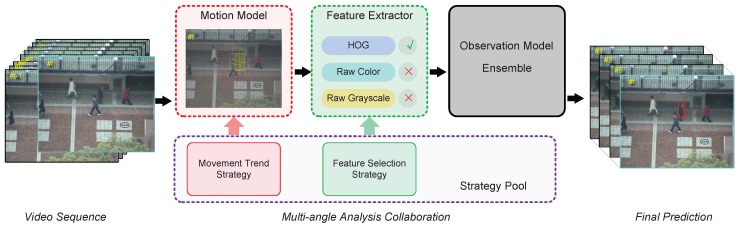
The overview of our Multi-angle Analysis Collaboration Tracking (MACT). By using the movement trend strategy, we can predict the position of the target in the motion model component, and then increase the weight of this part of the sample (the yellowish rectangle is the area where the target may appear, and the yellow dashed box is the high weight sample area). By exploiting the feature selection strategy, we can choose the current more appropriate image representation in the feature extractor component (current frame selection HOG feature). Through the mutual cooperation of the strategies obtained after multi-angle thinking, MACT enables more accurate and efficient tracking.

The overview of our multi-angle analysis collaboration tracking (MACT) is illustrated in Figure 1. The current frame is no longer subject to simple random sampling in the motion model component, but purposeful sampling and giving the corresponding region a higher weight under the guidance of the movement trend strategy. Unlike most tracking methods that use fixed image feature descriptions, in the feature extractor component, the feature of the image is dynamically selected by the corresponding feature selection strategy.

In summary, MACT’s main contributions are:

1. We confirm that tracking research should focus on the nature of the tracking problem, not just the classification method or network structure.
2. We propose a strategic framework that forms a one-to-one correspondence between the details of different perspectives and the action space of multi-dimensional state–action space reinforcement learning. This strategic framework can be extended according to different tracking tasks.
3. We obtain strategies from multi-angle analysis with reinforcement learning and apply them to specific traditional tracking frameworks.

Finally, we validated MACT on the public dataset OTB50 [1]. The experimental results show that MACT effectively improves the speed and accuracy of tracking.

## 2. Related Work

Visual tracking has been fundamental research in the field of computer vision over the past decade. As surveyed in [4,5], many researchers have achieved amazing results in mainstream visual tracking benchmark [1,6,7,8,9].

### 2.1. Visual Object Tracking

Traditional visual tracking algorithms are usually divided into two categories [10]. One approach constructs a generative model with previous experience to find the most matching area in the next frame. The other utilizes a discriminative model to separate the target from the background.

Generative tracking algorithms focusing on the targets description have received extensive attention in early tracking research. For example, Meanshift tracker [11], which is a tracking method based on probability density distribution, makes the search always follow the direction of the rising probability gradient, and iteratively converges to the local peak of the probability density distribution; Particle Filter tracker [12] is a method based on particle distribution statistics, which models the tracking object and defines a similarity measure to determine the similarity of the particle to the target; Kalman Filter tracker [13] is used to describe the motion model of the object for estimating the position in the next frame; and DRLTracker [14] models targets and backgrounds separately for collaborative tracking.

Discriminative approaches for visual tracking use the target as the foreground and make the online learning or offline training detector to distinguish the foreground object from the background. The main representatives of the tracking by detection method are: TLD [15], which applies multi-level classifiers to improve detection capabilities; and Struck [16], which uses structured SVM methods for online learning. It is worth mentioning that Martins et al. proposed a kernel tracking method, CSK, based on cyclic matrix, and solved the problem of dense sampling mathematically [17]. Some excellent improved correlation filter based tracking algorithms have been proposed, such as Kernelized Correlation Filters tracker (KCF) [18] and Discriminative Scale Space Tracker (DSST) [19].

For tracking based on deep learning, on the one hand, because the deep learning network model trained by big data can provide a more expressive feature representation, deep learning techniques are also widely used in computer vision research, including visual tracking research. In the early deep learning tracking research, the researchers directly integrated the features learned by the network into the relevant filtering or other tracking framework to obtain better tracking results, such as the DeepSRDCF [20]. Although this complex feature is expressed better than HOG or other conventional image features, it also brings a large amount of computation. Therefore, in later research, it is common practice to combine common features with depth features. These methods typically use common features in simple tracking scenarios and select depth features in complex tracking scenarios, such as C-COT [21] and ECO [22]. On the other hand, another major advantage of deep learning is the end-to-end output, which allows multiple tasks to be trained together, especially combining image feature networks with detection classification networks, which is suited for tracking research. Representative tracking methods include: GOTURN [23], SiameseFC [24] and CFNet [25].

### 2.2. Visual Tracking with Reinforcement Learning

Reinforcement learning is a learning mechanism that simulates the learning behavior of humans and higher animals. It emphasizes the constant “trying mistakes and improvements” in the interaction with the environment. As an important method in machine learning, reinforcement learning learns the optimal strategy of dynamic systems by perceiving environmental state information [26]. It enables expert-free online learning without a specialized system model.

At present, several scholars have applied reinforcement learning to the field of visual tracking. However, these applications are mostly limited to the improvement of the method, such as using reinforcement learning to mine deep expressions of deep neural networks. Specifically, Yun et al. [27] controlled the tracking strategy through actions that are trained by deep reinforcement learning. Zhang et al. proposed a fully end-to-end approach to predict the bounding box position for the object. They formulated tracking model as a recurrent convolutional neural network agent that interacts with a video over time [28]. Huang et al. used an adaptive approach to tracking with deep feature cascades and developed adaptive tracking issues as a decision process [29].

However, the core of reinforcement learning is to imitate human learning behavior, and the essence of tracking research is a simulation of human behavior. Therefore, different from the above methods, we use reinforcement learning to simulate the different perspectives of people, namely the strategic framework. Further, we use the independent strategic framework to guide the tracking framework.

## 3. Our Method

In this section, we divide the multi-angle analysis collaboration tracking (MACT) into two parts, the tracking framework and the strategic framework. The former consists of a basic tracking model [2], and the latter is implemented by a multi-dimensional state–action space reinforcement learning framework.

### 3.1. Tracking Framework with Basic Tracker

In our MACT tracker, the tracking model is only responsible for the basic tracking process, so the tracking framework only has basic tracking capabilities. Our tracking framework is inspired by the basic tracker proposed by Wang et al. [2], and consists of five parts: motion model, feature extractor, observation model, model updater and ensemble post-processor.

From the analysis in the Introduction, it can be seen that the motion model and feature extractor, respectively, correspond to the movement trend, which is from the angle of observer’s attention, and feature selection, which is inspired by object’s motion angle. In the basic tracker [2], the feature extractor component selects only one fixed feature representation (HOG feature), and the motion model component usually uses a sliding window to simply consider all possible candidates within the square neighborhood.

Different from the above two methods, to match the implementation of the strategic framework, we make some changes as follows.

- For the feature extractor component in MACT, multiple feature (HOG, Raw Color and Raw Grayscale) selections replace fixed single feature.
- For the motion model component in MACT, the selection of possible candidates evolves from random screening to purposeful selection.

According to Wang et al. [2], the overall performance of the HOG feature is superior to other features (raw color and raw grayscale) in the tracking research.

For the other three components, we use the most basic methods available in [2]:

- First, for the observation model component, we use the simplest logistic regression with $l_{2}$ regularization, and only employ the simple gradient descent to achieve online update of the model.
- Second, for model updater component, we adopt the common practice of setting a threshold [30]. The model is updated when the difference between the confidence of the target and the confidence of the background is below the threshold.
- Finally, for the ensemble post-processor component, we consider the reliability of each tracker as a hidden variable with reference to the study by Wang et al. [31], and then solve the problem of determining the tracking result by a factorial hidden Markov model.

For a complete tutorial about the basic tracker, we refer the readers to [2] for details.

It can be seen that our tracking framework is a simple tracking method without the aid of the strategic framework. The purpose of this design is to prove that ordinary tracking can be greatly improved after having multi-angle analysis cooperation.

### 3.2. Strategic Framework with Reinforcement Learning

**Figure 2:**
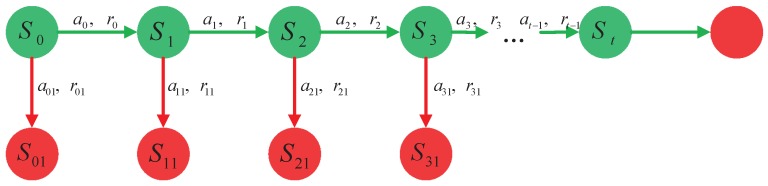
A simple decision model to track failures or successes. The solid green circle indicates successful tracking, and the solid red circle indicates tracking failure or end.

Unlike conventional tracking methods, MACT designed a meaningful strategic framework to guide the basic tracking framework described above. As shown in Figure 2, we treat the tracking process as a Markov Decision Process (MDP), and the agent can make a series of more reasonable and effective actions for motion model and feature extractor. This agent can predict where the target might appear and learn how to choose the appropriate image representation in the current frame. We treat the agent to learn the corresponding strategies by reinforcement learning. Therefore, we need to design the exclusive states, action space, and reward function for reinforcement learning.

#### 3.2.1. States and Action Space Design

Based on the above analysis, we know the learning model is defined by a Markov Decision Process (MDP). The MDP is a 4-tuple model:

(1) 〈S,A,T,R〉

For simple verification, in our study, the state *S* is defined by the time frame. During the training phase (see Equation (2)), there are two possible states for each frame: judgment of tracking result (tracking success or tracking failure) and image similarity of the target τ. In the operational phase, there is only one state: image similarity, as shown in Equation (3).

(2) $$S_{t}=\left\{success,failure\right\}\times \left\{\tau \right\}$$

(3) $$S_{o}=\left\{\tau \right\}$$

where $\tau_{t}$ denotes the image’s similarity hash vector of the current frame in the *t*th state, and its calculation algorithm is shown in [32]. $h_{t}$ is mean value of historical similarity hash.

(4) $$\tau_{t}=\frac{\sqrt{\sum {\left(h_{t}-{\bar{h}}_{t}\right)}^{2}/t-1}}{{\bar{h}}_{t}}$$

In addition, to reduce the amount of calculation, MACT defines a state every five frames.

In the Introduction, we propose to link the angle of thinking to the action space. Therefore, in MACT, we implement two tracking analyses (the observer’s attention and the object motion) in the multidimensional action space. Specifically, the observer’s attention corresponds to this feature selection and the object’s motion corresponds to the movement trend. Here, we only use three features (raw color, HOG and raw grayscale) and four directions (up, down, right and left). Thus, the definition of the action space **A** for any state in *S* is as follows:

(5) $$A=\left\{↑,↓,\to ,\leftarrow \right\}\times \left\{RawColor,HOG,RawGrayscale\right\}$$

Obviously, the state transition function is defined as follows:

(6) T:S×A×S→[0,1]

The reward function R:

(7) R:S×A→R

Thus, the strategy (or policy) is denoted by $\pi :S\times A\to \left[0,1\right]$ which maps states and actions to a probability. The probability of choosing an action *k* according to policy π is π(k). A strategy is deterministic or pure if the probability of playing one action is 1, while the probability of playing other actions is 0 (i.e., ∃π(k)=1 AND ∀l≠k,π(l)=0), otherwise the strategy is stochastic or mixed.

The goal of a reinforcement learning algorithm is to find a strategy for every state in *S* to optimize the expected reward, which is defined by long-term expected reward of the policy. Formally, it has two representations: the state value function,

(8) $$V_{\pi }\left(s\right)=E_{\pi }\left[r^{t+1}+\gamma r^{t+2}+\gamma^{2}r^{t+3}+\ldots |S_{t}=s\right]$$

and the state–action value function,

(9) $$Q_{\pi }\left(s,a\right)=E_{\pi }\left[r^{t+1}+\gamma r^{t+2}+\gamma^{2}r^{t+3}+\ldots |S_{t}=s,A_{t}=a\right]$$

where γ is the discount factor.

Further, this strategy can be divided into feature selection strategy (FSS) and movement trend strategy (MTS) according to different dimension actions (feature selection and direction selection).

#### 3.2.2. *Q*-learning and Exploration Strategy

Considering maturity and reliability, we use *Q*-learning to find the optimal policy in this work [33]. The *Q*-learning algorithm is a classical value function-based reinforcement algorithm. Because it does not need to establish an environment model and guarantees convergence under certain conditions, it is the most widely used algorithm in reinforcement learning. The main steps of *Q*-learning are summarized in Algorithm 1.

**Algorithm 1***Q*-Learning: An Off-policy TD Control Algorithm
Initialize Q(s,a), ∀s∈S, a∈A(s), arbitrarily, and Q(terminal−state,·)=0Repeat (for each episode):     Initialize *S*     Repeat (for each step of episode):        Choose **A** from *S* using policy derived from *Q* (e.g., ϵ−greedy)        Take action **A**, observe *R*, $S^{\prime }$        $Q\left(S,A\right)\leftarrow Q\left(S,A\right)+\alpha [R+\gamma \max_{\alpha }Q\left(S^{\prime },a\right)-Q\left(S,A\right)]$        $S\leftarrow S^{\prime }$     until *S* is terminal


The core of *Q*-learning is the *Q*-table. The rows and columns of the *Q*-table represent the values of the state and action, respectively. The *Q*-table value Q(s,a) records the estimation of the state–action value function $q^{\pi }\left(s,a\right)$. During the training process, the algorithm uses the ϵ-greedy [34] exploration strategy to select actions. The ϵ-greedy exploration strategy is an improvement of the greedy strategy, which refers to the form of the probability distribution when making action selections.

Under the current state *s*, the agent selects randomly by probability ϵ, which is called the random exploration process, and selects the action which has the max *Q*-value with the probability 1−ϵ, which is called the exploitation process. Another famous exploration strategy is the Boltzmann exploration strategy, which can use the value function to dynamically adjust the balance between exploration and utilization in action selection.

Considering the fast convergence requirements of the model, we use ϵ-greedy in our model. After selecting the action, the agent observes the return *r* and the next state $s^{\prime }$ from the environment, and then the *Q* algorithm uses the Bellman Equation to update the *Q*-table, ie.,

(10) $$Q\left(s;a\right)=r+\gamma (Max\left(Q\left(s^{\prime };a^{\prime }\right)\right))$$

The Bellman equation can be understood as: Q(s;a) is expressed as the immediate return *r* after taking *a* in the current *s*, plus the maximum expected return $Max(Q\left(s^{\prime };a^{\prime }\right))$ after the discount γ. The Bellman equation, also known as the Dynamic Programming Equation, is a necessary condition for mathematical optimization methods, such as dynamic programming, and is also a basic concept for multi-state problems in the reinforcement learning.

#### 3.2.3. Reward Function Design

During the training process, the agent gets a reward *r* based on an action *a* in the current state *s*. It is closely linked to specific tasks. A good reward function not only speeds up the learning process, but also increases the value of decision making. In MACT, rewards *r* consists of three parts: tracking quality reward $r_{tq}$, feature selection reward $r_{fss}$, and movement trend reward $r_{mts}$.

(11) $$r=r_{tq}+r_{fss}+r_{mts}$$

In particular, $r_{tq}$ is not only part of rewards *r*, but also determines the scores of $r_{fss}$ and $r_{mts}$. $r_{tq}$ is defined as:

(12) $$r_{tq}=\left\{\begin{array}{c} \begin{array}{ccc} +10 & if & IoU\geq t_{iou} \end{array}\\ \begin{array}{ccc} -10 & otherwise & \end{array} \end{array}\right.$$

In particular, The definition of IoU in Equation (12) refers to the overlap score, which was defined by Wu et al. [1]:

(13) $$IoU=\frac{\left|p\cap g\right|}{\left|p\cup g\right|}$$

where ∩ and ∪ mean the intersection and union of two regions (*p* indicates the current object position and *g* represents ground truth bounding box), and $\left|\cdot \right|$ denotes the number of pixels in the region. We set the threshold $t_{iou}=0.5$ according to Wu et al. [1]. The definitions of $r_{fss}$ and $r_{mts}$ are related to $r_{tq}$ as follows:

(14) $$r_{fss}=\left\{\begin{array}{c} \begin{array}{ccc} r_{fss_{s}} & if & r_{tq}=+10 \end{array}\\ \begin{array}{ccc} r_{fss_{f}} & & otherwise \end{array} \end{array}\right.$$

(15) $$r_{mts}=\left\{\begin{array}{c} \begin{array}{ccc} r_{mts_{s}} & if & r_{tq}=+10 \end{array}\\ \begin{array}{ccc} r_{mts_{f}} & & otherwise \end{array} \end{array}\right.$$

In Equation (14), $r_{fss_{s}}$ and $r_{fss_{f}}$, respectively, represent the value of $r_{fss}$ when the tracking succeeds or fails. Similarly, the same definition applies to both $r_{mts_{s}}$ and $r_{mts_{f}}$. The specific score distribution scheme is shown in Table 1 and Table 2. Feature selection strategy Reward (*FSS Reward*) in Table 1 indicates the score obtained when the agent selects different feature expressions in different states, and movement trend strategy reward (*MTS Reward*) in Table 2 is the score corresponding to the action in different directions.

It is worth noting that, when the tracking framework selects the HOG feature, on the one hand, in the state of successful tracking, the rewards we designed are relatively low, and, on the other hand, in the state of tracking failure, the penalty is relatively high. The reason for this design is that, under the premise of ensuring effectiveness, MACT encourages the tracking framework to use simple and effective feature representation as much as possible to improve tracking efficiency.

Therefore, the goal of training agent is to maximize the sum of reward R throughout the video sequence:

(16) $$R=\sum r_{i}$$

#### 3.2.4. Mutual Cooperative

**Figure 3:**
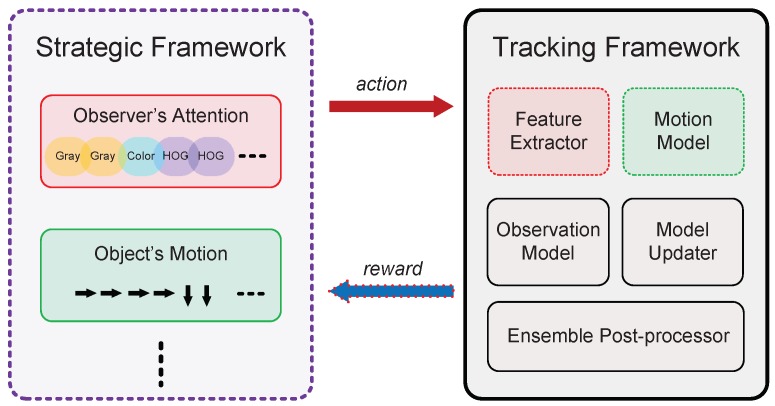
Schematic diagram of cooperation between the strategic framework and tracking framework.

The strategic framework in Section 3.1 and tracking framework in Section 3.2 together form our multi-angle analysis collaboration tracking (MACT). As illustrated in Figure 3, strategic framework models two thinking processess (observer’s attention and object’s motion), and of course it can continue to expand. After training by reinforcement learning, the strategic framework can obtain corresponding strategy. Specifically, our MACT adopts the feature selection strategy (FSS) to guide the motion module for purposeful sampling, and hires motion trend strategy (MTS) to choose a more appropriate image feature.

#### 3.2.5. Discussion

**Figure 4:**
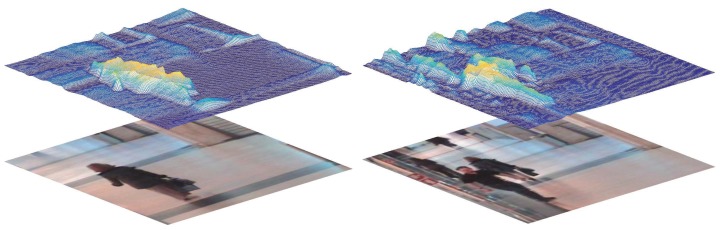
Visual saliency map reflecting visual attention under different conditions. The left column indicates a “clean" background (without similar disturbances) and the right column is a “complicated" background (with some similar and serious disturbances).

Psychological research has found that targets that are significantly different from the surrounding area are likely to attract the viewer’s visual attention [35], and the study of visual attention is visual saliency research [36]. Therefore, we believe that, in the tracking process, people are more likely to be attracted by the target when the background and target are more different (no similar disturbances around the target or the background does not clutter); otherwise, people need to find more differences between the target and the background. As shown in Figure 4, when there are no other pedestrians around the target (the walking woman), its saliency value (visual attention measure calculated in [36]) is quite obvious compared to the surrounding. Once there are some similar pedestrians nearby, the target’s saliency value is no longer obvious, even lower than the pedestrians.

Therefore, when the tracking environment is complex, the tracking process becomes difficult, and it is necessary to obtain more information to identify the target from the background. In the MACT, we analyze the tracking process from different angles to obtain different discriminating information.

**Figure 5:**
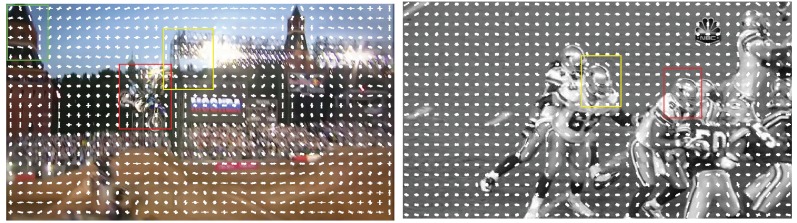
Visualization of HOG features in *MotorRolling* and *Football*. Rectangular boxes of different colors represent tracking results from different features: Red indicates HOG, yellow represents raw grayscale and green means raw color.

HOG feature is the last choice in MACT’s feature selection strategy (FSS). Raw grayscale feature simply converts the image to grayscale and then uses the pixel value as a feature. Although the processing method is simple, in some suitable tracking scenarios, this simple feature can achieve good tracking results. Raw color is basically the same as the raw grayscale except that the image is represented in the color space instead of the grayscale. This feature is significant when the object and the background are clearly distinguishable in color. However, when the above cases are not satisfied, the effects of raw grayscale and raw color are greatly reduced. Showing excellent overall performance [2], especially the ability to describe the local shape of the object [3], HOG is adopted by FSS. As shown in Figure 5, in the *MotorRolling* video sequence, raw grayscale and raw color cannot capture the target well due to factors such as illumination effects and target blur; in the grayscale *Football* video sequence, raw grayscale has difficulty coping with this situation, because of similar interferences around the target.

**Figure 6:**
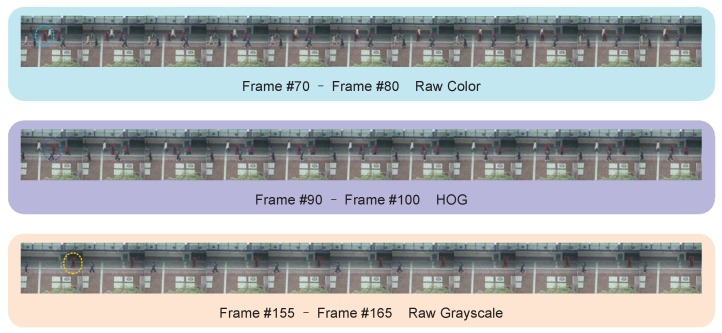
The example sequence of strategy taken to match the most appropriate features in the different current scene. Feature usage is determined by the corresponding option of maximum score on the score map. Our agent learns to wisely act upon the score maps. When the score maps are ambiguous, the agent postpones the decision and uses all features according to the more unambiguous score map at the next frame. Further selections of image feature are performed with more balance and stronger features confidence.

We use *Subway* to demonstrate the advantages of feature selection strategy. From the results shown in Figure 6, we observe that, from Frame 70 to Frame 80, there is only one passerby in white clothes near the object pedestrian. Because the color of their clothes is very different, it is easy to use color features to distinguish between object and interferers. Similarly, between Frame 90 and Frame 100, there is interference with similar colors around the object. At this time, the color features no longer have good discriminability. Under the guidance of the strategic framework, the tracking framework uses HOG feature with high computational complexity but strong expressiveness and better overall performance to perform feature processing. In the later stages of the video, there are basically no similar interferers around the target, i.e., the object and background are very different, therefore, simple grayscale features can achieve good tracking results. It can be seen that a good feature selection strategy can not only improve the tracking speed, but also improve the tracking accuracy to a certain extent.

**Figure 7:**
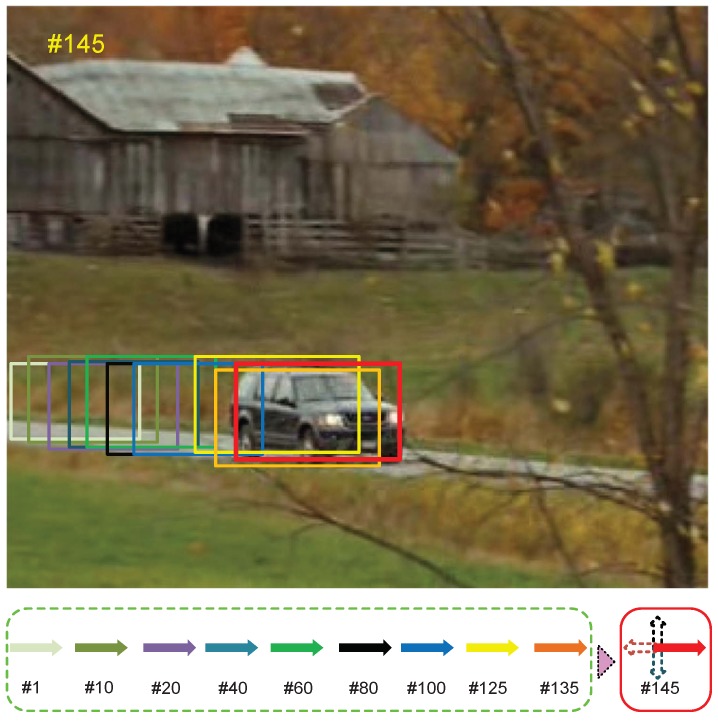
Movement trend diagram for the *CarScale* sequences. Rectangular boxes of different colors represent the object positions at different times. The red rectangle indicates the ground truth position in the #145 frame. Arrows of different colors indicate the movement trend of the object in different frames. The cross arrow indicates four directions about movement trend, where dotted arrows are the low probability directions.

Figure 7 shows the importance of the movement trend strategy. In the *CarScale* sequence, the car travels in one direction, and accurate motion trend estimation allows the motion model in the tracking frame to better select samples. Of course, in most tracking videos, the trajectory of the object is not determined. However, for a short period of time, the object’s movement trend is still predictable due to inertia.

## 4. Experimental Results

In this section, we validate our MACT tracker on the OTB50 dataset using CVPR2013 benchmark evaluation method [1], and then compare the test results with the current mainstream tracking methods. All experiments were performed on a personal computer: MATLAB R2014b, Intel i7-4790 CPU with 4G DDR3 memory.

### 4.1. Dataset and Evaluation

OTB50 is a small and complete dataset which contains 50 fully annotated video sequences: *tiger2, tiger1, subway, football, basketball, faceocc2, faceocc1, woman, liquor, lemming, mountainBike, motorRolling, suv, dog1, skiing, carScale, jumping, david3, freeman4, freeman3, freeman1, fleetface, walking, walking2, girl, doll, jogging2, jogging1, football, couple, crossing, dudek, boy, bolt, coke, mhyang, fish, trellis, sylvester, david2, david, car4, carDark, singer2, singer1, shaking, skating1, deer, ironman,* and *soccer* [1]. These video sequences basically cover all the challenges in tracking tasks, including illumination variation (IV), scale variation (SV), occlusion (OCC), deformation (DEF), motion blur (MB), fast motion (FM), in-plane rotation (IPR), out-plane rotation (OPR), out-of-view (OV), background clutters (BC), and low resolution (LR) [1].

To facilitate tracking evaluation, we employed the classic CVPR 2013 benchmark evaluation system. The evaluation method for each frame consists of two indicators: precision plot and success plot. The former is defined as the average Euclidean distance between the center locations of the tracked targets and the manually labeled ground truths, while the latter shows the ratios of successful frames as the threshold varies from 0 to 1 [1]. For the robustness evaluation of a video sequence, we adopted one-pass evaluation (OPE), which is the average accuracy or success rate of the entire video sequence after running according to ground truth position directly (see [1] for details).

### 4.2. Strategic Framework Test

To validate the effectiveness of the policy framework, as shown in Table 3, we compared MACT with three specially designed trackers. The Basic tracker is basically consistent with the tracking framework of MACT (see Section 3.1 for details), the difference being that the former uses random sampling and fixed features HOG. MACT_FSS tracker is consistent with MACT, except that the strategic framework only contains feature selection strategies (FSS), and its motion model uses the same random sampling as the Basic tracker. Finally, MACT_MTS is similar to MACT but removes the feature selection strategy and adopts HOG feature in feature extractor.

**Figure 8:**
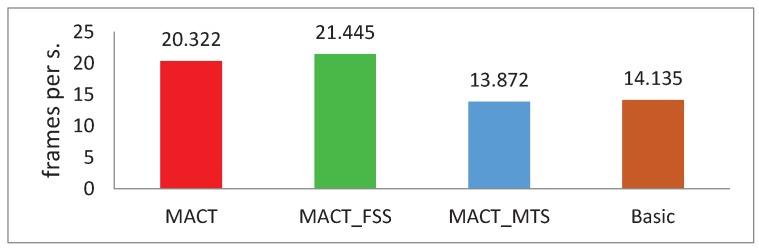
Comparison of the running speeds of four tracking methods on the OTB50 test set under the same test environment.

We experimented with these four trackers on the OTB50 over all 50 videos. On the tracking speed indicator, we compared these four methods, as shown in Figure 8. MACT_FSS tracker has the best tracking speed (21.445 frames per second), MACT is 5.23% slower than the best speed. The reason is that MACT_FSS does not need to predict the target motion trend, which also shows that the impact of motion trends on tracking speed is very small. Due to the large amount of HOG feature calculation, the tracking speeds of the other two trackers are obviously much lower.

**Figure 9:**
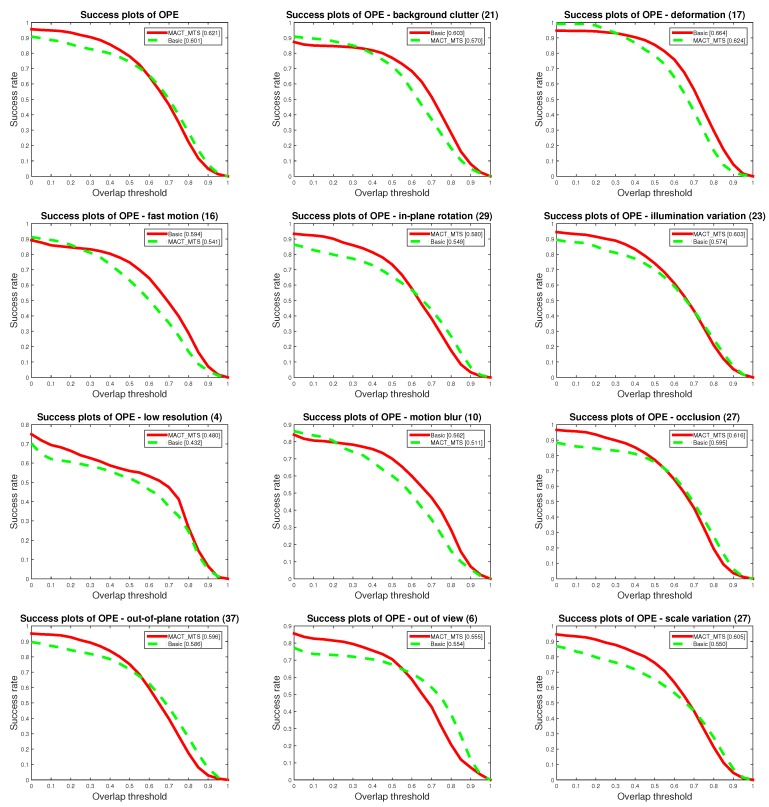
Comparison of MACT_MTS tracker and Basic tracker on OPE protocol with success plots.

**Figure 10:**
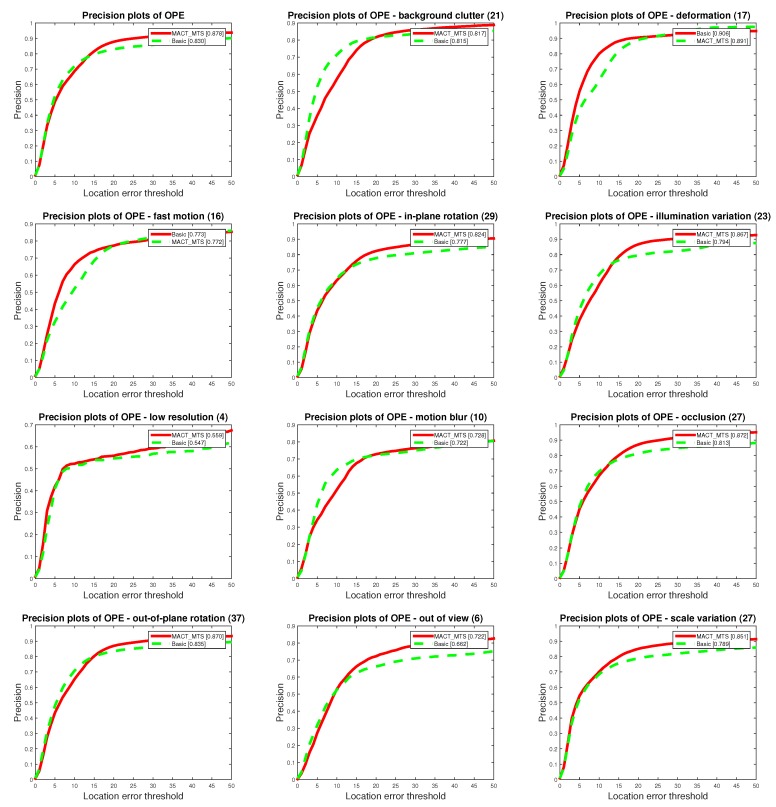
Comparison of MACT_MTS tracker and Basic tracker on OPE protocol with success plots.

To better understand the improvement of tracking accuracy by the strategic framework, we compared MACT_MTS tracker with the Basic tracker to verify the validity of the movement trend strategy. As shown in Figure 9 and Figure 10, MACT_MTS’s OPE protocol score is superior to Basic tracker in both success plot and precision plot, and the MACT_MTS had the better score for most of 11 different attributes (background clutter, out-of-plane rotation, illumination variation, in-plan rotation, motion blur, fast motion, deformation, occlusion, out of view, low resolution, and scale variation). This shows that the movement trend strategy (MTS) can improve the accuracy of tracking.

**Figure 11:**
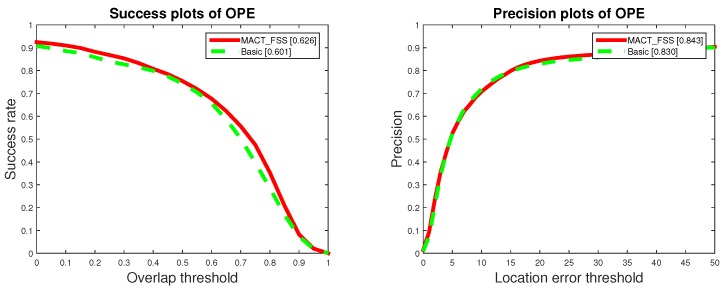
Overall comparison of MACT_FSS tracker and Basic tracker on OPE protocol.

**Figure 12:**
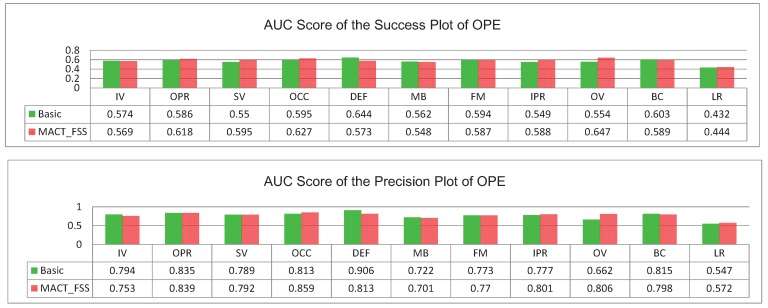
Detailed AUC scores on 11 attributes on OPE protocol.

For the validity verification of the feature selection strategy (FSS), we chose the MACT_FSS tracker to compare with the Basic tracker. As shown in Figure 11 and Figure 12, we found that, with the guidance of the FSS, the accuracy of the MACT_FSS tracker has been greatly improved; specifically, the OPE success plot score increased by 4.16%, and the precision AUC score increased by 1.57%.

**Figure 13:**
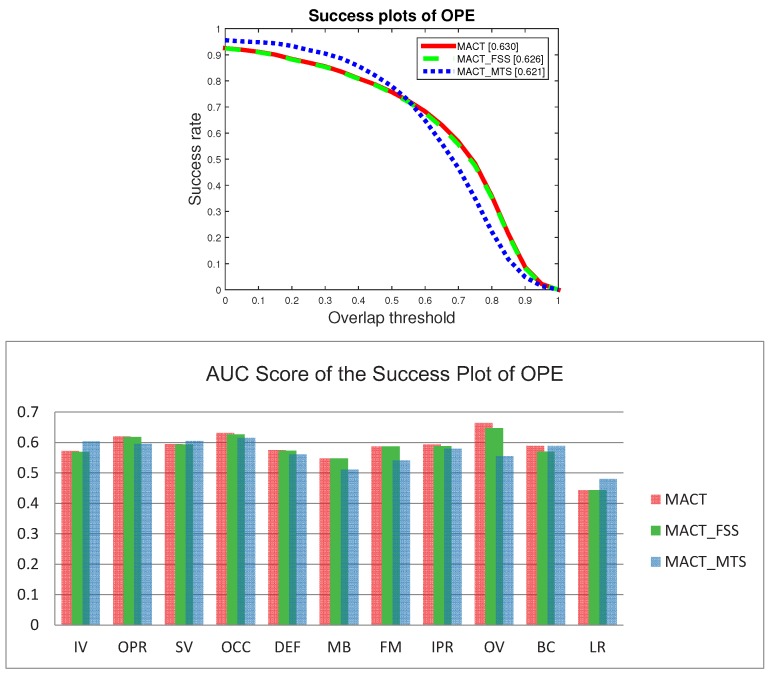
One Pass Evaluation (OPE) plots on OTB. The top row is the success plots for MACT tracker, MACT_FSS tracker and MACT_MTS tracker with the overall scores in the legend, and the bottom row demonstrates the AUC scores for over 11 attributes.

The experiment of the single strategy proves that FFS can not only greatly improve the tracking speed, but also improve the tracking accuracy; although MTS has a slight influence on the tracking speed, it can also improve the tracking accuracy. When these two strategies act on the strategic framework at the same time, that is our MACT tracker, the experimental data prove that the cooperation between FSS and MTS improves both the speed (compared with the MACT_MTS) and the accuracy, as shown in Figure 13.

Although MTS reduces the tracking speed, it has achieved the goal of jointly improving tracking accuracy in cooperation with FSS. Therefore, we believe that the computational burden of MTS is acceptable.

### 4.3. MACT Tracker Test

After verifying the effectiveness of the strategy, we compare the performance of the MACT with the other tracking methods in two aspects: quantitative comparison and qualitative comparison.

#### 4.3.1. Quantitative Comparison

Since MACT is based on the traditional tracking framework, its most expressive feature is basic HOG. Therefore, our comparison is limited to the mainstream tracking methods using traditional features, including traditional peaking trackers (DSST [19] and KCF [18]) and 29 tracking methods in the benchmark 2013 [1] (SCM [37], TLD [15], Struck [16], ASLA [38], CXT [39], VTD [40], VTS [41], DFT [42], CPF [43,44], OAB [45], LSK [46], MTT [47] and so on).

**Figure 14:**
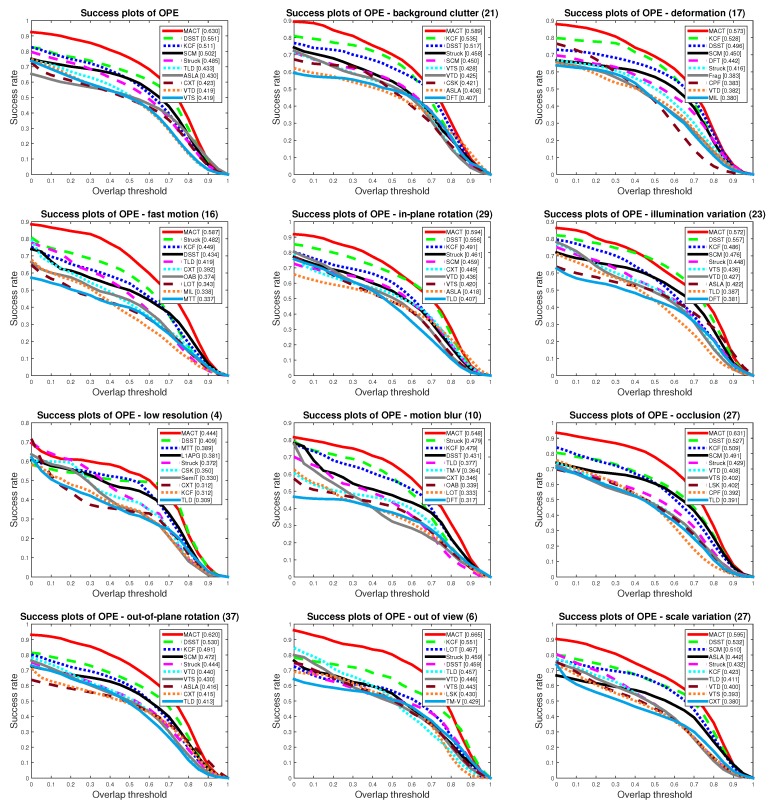
Comparison of Ttop 10 trackers on OPE protocol with success plots.

**Figure 15:**
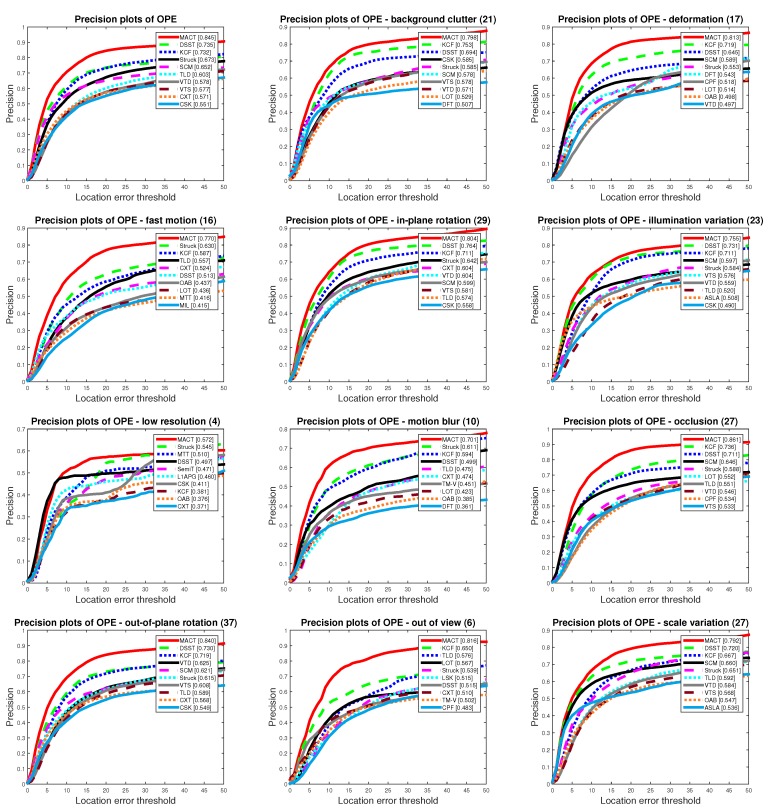
Comparison of the Top 10 trackers on OPE protocol with precision plots.

As can be seen from the success and precision plots of OPE in Figure 14 and Figure 15, our MACT has considerable advantages. In 11 attributes, our method is mostly leading the other trackers.

In Table 4, we compare MACT with four representative state-of-the-art competitors in VOT-2015 [48]. Although MACT has lower accuracy score than MDNet [49] and Staple [50], it runs much faster than the other four trackers in terms of normalized speed. Compared with DSST [19] and MEEM [51], MACT leads in all three indicators. Without sophisticated optimization strategies and high-precision feature representation, MACT still gets good tracking performance.

It can be seen that, through the assistance of the strategic framework, MACT is basically in the lead position among the traditional tracking methods.

#### 4.3.2. Qualitative Comparison

Since MACT is superior to most traditional tracking methods, we directly chose to compare with MUlti-Store Tracker (MUSTer) [52] in a qualitative comparison. MUSTer’s concept is very clever (a dual-component: short-term memory and long-term memory store), and the features selected are more complicated: 31-dimensional HOG descriptors. Because of its clever design, more expressive features, and excellent program optimization, MUSTer is the leader among current tracking methods based on non-depth feature descriptions.

**Figure 16:**
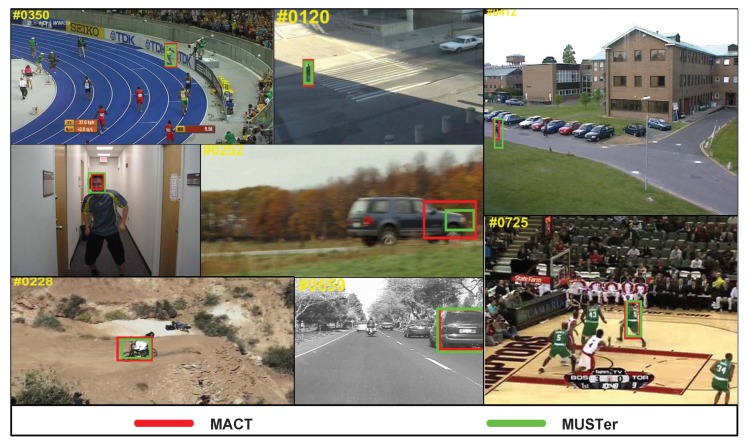
Tracking results on several classic examples.

On the OTB50 dataset, we tested both the MACT and MUSTer methods. MUSTer’s overall performance is even better. As shown in Figure 16, both methods have a good performance in most video sequences, such as *basketball, bolt, boy, car4, carScale, crossing, mountainbike, walking*, etc.

**Figure 17:**
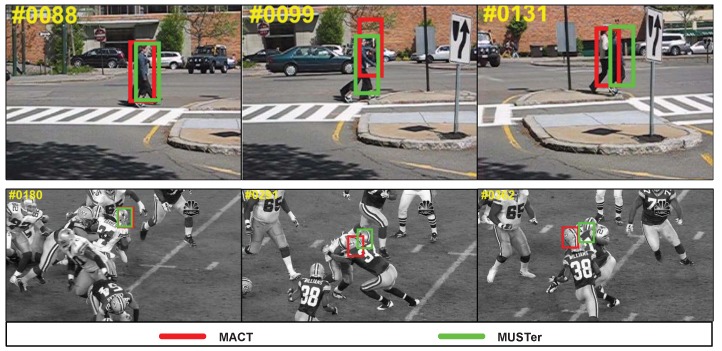
Example of tracking results with MUSTer advantages.

Figure 17 shows that MUSTer performs well in some challenging video sequences. Specifically, in the *couple* video sequence, in the #1–#90 frame period, the two methods can capture the object, and in the #99 and #100 frames, the target is severely occluded and the interference is consistent with the target motion trend. The MACT drifted due to the lack of more precise feature options and corresponding processing mechanisms. In addition, in the *football* video sequence, when there is a very similar interference around the object player, MACT begins to show poor performance, although MACT has a movement trend strategy.

**Figure 18:**
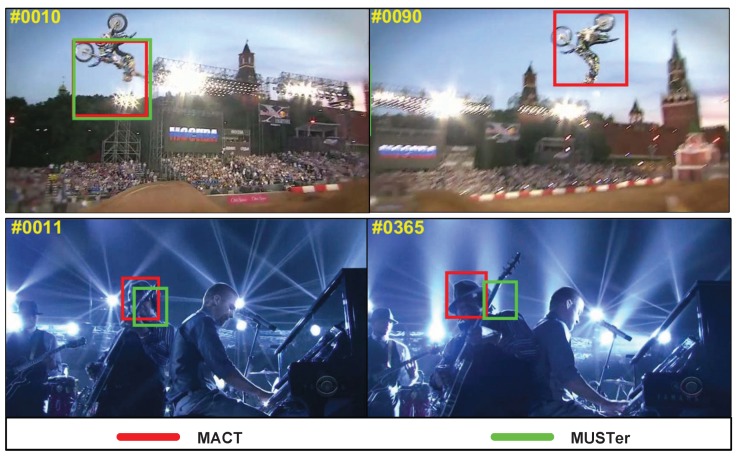
Example of tracking results with MACT advantages.

Although MACT is slightly inferior to MUSTer, we found that MACT has certain advantages in other challenging video sequences. As shown in Figure 18, in the *motorRolling* video, the object is a motorcycle. The video’s difficulty is that the object speed is extremely fast and the number of video frames is very small, and the feature at this video is relatively not a key point, so MUSTer does not have an advantage, but MACT shows strong robustness because of the guidance of the movement trend strategy (MTS). The same situation occurs in another video sequence (*shaking*); the gray objects have similar colors to dark background, so the color and gray features are not well discriminative. In addition, there are similar interferences around the target (for example, piano and guitar players), so the HOG feature does not have an advantage. At this time, the MACT can correct the target drift, due to the guidance of the movement trend strategy (MTS).

## 5. Conclusions

In this paper, we incorporate a novel strategic framework on traditional tracking framework based on multi-angle analysis collaboration. We believe that visual tracking research should not only consider machine learning methods or deep neural network, but also really need to think about the nature of tracking problem from different perspectives.

In our method, two thinking angles, namely observer’s attention and object’s motion, are selected from a simple case study, enabling them to better handle tracking. Specifically, we choose the image feature to implement the observer’s attention angle, and adopt the object’s movement trend to reflect the object’s motion angle. Selection of suitable image features and prediction of current object movement trend are determined by strategy pool in the strategic framework. It is worth noting that the type of features and the direction of the movement trend correspond one-to-one with the actions in the action space. The learning of strategy is completed by the *Q*-learning and ϵ-greedy exploration in reinforcement learning. Obviously, our MACT tracker is a fusion of the clever strategic framework and the basic tracking framework.

Experiments over the OTB50 benchmark demonstrate that our MACT tracker achieves a high evaluation and avoided drift to some extent. The motivation for the paper is simple: return tracking research to thinking about tracking behavior. Mapping states in the multi-dimensional state–action space and tracking thinking from different angles can help to solve tracking problems in different tracking environments and thinking modes. 

## Figures and Tables

**Table 1:** FSS reward corresponding to different feature selection actions.

FSS Reward $r_{fss}$	Raw Color	HOG	Raw Grayscale
tracking success: $r_{fss_{s}}$	10	8	10
tracking failure: $r_{fss_{f}}$	−1	−5	−1

**Table 2:** MTS reward corresponding to different direction selection actions.

MTS Reward $r_{mts}$	Up	Down	Right	Left
tracking success: $r_{mts_{s}}$	10	10	10	10
tracking failure: $r_{mts_{f}}$	−5	−5	−5	−5

**Table 3:** Details of the strategies included in the four tracking methods

	MACT	MACT_FSS	MACT_MTS	Basic
feature selection strategy (FSS)	Yes	Yes	No	No
movement trend strategy (MTS)	Yes	No	Yes	No

**Table 4:** Comparative results on VOT-2015 dataset.

Trackers	Accuracy	Overlap	Speed
MDNet	0.5607	0.3489	0.7928
Staple	0.5339	0.2651	10.5469
**MACT**	**0.5099**	**0.2107**	**14.613**
DSST	0.5071	0.1663	6.7001
MEEM	0.4811	0.2083	4.8127
